# Violence Exposure, Mental Health, Cognitive Functioning, and Disabilities in Incarcerated Youth

**DOI:** 10.21203/rs.3.rs-8436385/v1

**Published:** 2026-01-20

**Authors:** Suzanne Perkins, Sriamsha Dubbaka, Erin Wang, Michaela Wu, Wendy Su, Vanessa Assaf, Julianne Park, Joanne Smith-Darden, Sandra Graham-Bermann

**Affiliations:** University of Michigan–Ann Arbor; Lurie Children’s Hospital; University of Michigan–Ann Arbor; University of Michigan–Ann Arbor; University of Michigan–Ann Arbor; University of Michigan–Ann Arbor; University of Michigan–Ann Arbor; Michigan State University; University of Michigan–Ann Arbor

**Keywords:** Violent offenders, Juvenile Justice, Comorbid Diagnosis, Executive Functioning, Adverse Childhood Experiences

## Abstract

**Objective.:**

The goal of the present study was to ascertain incidence rates of violence exposure (VE) and disabilities in a population of incarcerated male adolescents using records searches, survey research, and individual academic and cognitive testing.

**Method.:**

A sample of 115 incarcerated male youth completed self-reported measures of VE, including adverse childhood experiences (ACE), child abuse and neglect, interpersonal Violence experiences (IPV), and community violence exposure (CV), and current functioning and individual testing of cognition and disability diagnosis (both cognitive and mental health).

**Results.:**

Two-thirds of the participants reported high ACE, 90% experienced at least mild physical abuse, and one-third reported severe prior sexual abuse (SA) using the CTQ. Over 60% had a prior disability in their records, and over 75% were diagnosable during individual cognitive testing. Just over 50% of youth were diagnosable with a mental health diagnosis, and with cognitive disabilities, the incidence rate increased to 85% of the population. Youth with disabilities had higher rates of VE.

**Conclusion.:**

Few studies combine data related to prior VE, disability diagnosis using individual measures, and test for current cognitive functioning in incarcerated youth. The present study determined that high rates of disability were largely driven by cognitive and academic disabilities, rather than mental health problems, indicating a pervasive problem with cognitive disabilities in youth who are incarcerated. Cognitive disabilities, particularly in youth with prior histories of VE, are an intervention point that has the potential to reduce recidivism.

## Introduction

Violence exposure (VE) is a national problem that is particularly salient for incarcerated youth. The Crime Against Children Research Center found that 4 in 10 youth (41.2%) from a nationally representative sample had experienced at least one incident of physical assault in the past year, and approximately 40% had witnessed violence at some point in their lives in the United States (Finkelhor et al., 2011)

Research indicates that a significant proportion of delinquent youth were exposed to violence during key developmental stages. In one study, 60% reported having experienced child abuse or neglect (Moore et al., 2013). Dysfunctional family dynamics are another factor that commonly impacts incarcerated youth, and with VE are collectively called adverse childhood experiences (ACE). One study found that in young adults with official criminal records for crimes committed between the ages of 12–15, parental separation, physical abuse, and incarcerated household members were found to be significant predictors for juvenile delinquency, and incarcerated household members were found to be a predictor for crime Persistence (Daquin et al., 2023).

VE is a significant risk factor for poor mental health outcomes and engagement in risky behaviors (Annett et al., 2023; Henry, 2020), such as substance use, violent behavior, and attitudes towards aggression, such as increased support for aggressive attitudes, self-reported aggression, and finally lifetime violent offending (Ball, 2005; Moore et al., 2013). Mental health issues are also more prevalent among incarcerated youth, with nearly 8 in 10 incarcerated youth diagnosed with at least one psychiatric disorder (Beaudry et al., 2021), which has led to theories of VE leading to later offending, such as the cycle of violence (Ball, 2005). One often overlooked factor associated with youth incarceration is high rates of disabilities (Mallett et al., 2023). In fact, Mallett and colleagues found that children who have received services for learning disabilities have an increased risk of crime and delinquency, and that leads to an increased rate of juvenile incarceration. A recent Canadian study found that disabilities were more than double in the adult incarcerated population (Whittingham et al., 2020). While another study found that of the 66% of inmates reported having a disability, 40.4% had non-psychiatric disabilities, which consisted of cognitive disabilities, ADHD, or enrollment in “special education”, indicating this as an important area to investigate (Bixby et al., 2022).

Most studies of disabilities in incarcerated populations rely of records searches or self-reports to determine disability status, which may over- or under-report cases (Young et al., 2015). Few previous studies of incarcerated youth have used diagnostic tools to assess cognitive and academic functioning (Chow et al., 2022; Fazel et al., 2008; Young et al., 2015). Additionally, most studies of disabilities in incarcerated populations include only one sub-area of disability, such as ID (Fazel et al., 2008), LD (Chow et al., 2022), or ADHD (Young et al., 2015). In fact, meta-analytic and systematic reviews on disabilities are limited because researchers have done a poor job of characterizing multiple disabilities in incarcerated populations, particularly with diagnostic testing. To understand how high rates of VE, mental health diagnosis, and cognitive and academic disabilities may interact to lead to violent offending, it will be important to understand comorbidity in juvenile justice populations. To develop targeted interventions for this population, it will be necessary to understand how disability and VE interact among young offenders.

The goal of the present study was to ascertain incidence rates of VE and both physical and mental health disabilities in a population of incarcerated male adolescents using records searches, survey research, and individual academic and cognitive testing. The first step in understanding potential causation is to chronicle the scope of the problem.

## Method

### Participants

Participants were recruited from an all-male youth incarceration facility in southeastern Michigan and included all willing participants. Human Subjects approval was granted by the University of Michigan Institutional Review Board in consultation with the State Office of Human Services, which approved the facility’s cooperation. A federal certificate of confidentiality was obtained from the National Institutes of Health to protect participant data. Further details on recruitment and consent procedures have been previously reported (Author reference, 2011; Author reference, 2012). Data from the study are publicly available through the University of Michigan Inter-university Consortium for Political and Social Research (Author et al., 2023).

### Record Review

The review of medical, educational, and intake records was carried out by trained graduate and undergraduate students. The records were coded for medical and educational diagnoses and criminal offenses. More information on the collection of each of these records can be found in the supplemental (see Supplemental Text 1).

### Survey Methods

Participants completed a survey booklet and two validated self-report measures of functioning. The booklet included demographic measures, family and educational history, and 11 validated scales. The validated scales included measures of violence exposure, delinquency, prior offending, family functioning, and mental health functioning.

#### Violence Exposure.

Violence exposure was assessed with four well-known measures of children’s exposure to violence in the domains of adverse childhood experiences (ACE), childhood experiences of trauma, interpersonal Violence in the family (IPV), and community violence exposure (CVE).

#### Adverse Childhood Experiences (ACE).

ACE was measured with 13 items. Questions included criminality in the family, parental use and trafficking in alcohol/drugs, childhood neglect, physical and sexual abuse, interpersonal violence in the family, and poverty. A total score was obtained.

#### Childhood Trauma Questionnaire.

A modified 34-item Childhood Trauma Questionnaire (Bernstein et al., 1994) was used to measure the experience of various types of childhood abuse and neglect, including emotional abuse, emotional neglect, physical abuse, physical neglect, and sexual abuse. It was assessed on a 5-point Likert scale (1 = never true, 2 = rarely true, 3 = sometimes true, 4 = often true, 5 = very often true). Sub-scales were created of emotional abuse and neglect, physical abuse and neglect, and sexual abuse. Recognizing that youth may often misinterpret sexual abuse behaviors in unhealthy relationships as “dating” and expressions of affection, an additional question was added to the sexual abuse sub-scale: “I had sex with an adult or with someone a lot older than me (someone at least 5 years older than me).”

#### Interpersonal Violence (IPV).

IPV was assessed with a modified version of the Conflict Tactics Scale (CTS-modified), (Straus, 1979) which was used to measure the frequency and severity of conflict-related violence within the family. The version used in this study was modified to include violence experienced by the youth from siblings. A total of 18 items were assessed under four sub-scales of violence: Personal Violence, Sibling Violence, Parental Violence, and Interpersonal Violence. The Personal Violence sub-scale evaluates the individual’s experiences of physical and psychological violence directed towards them by their siblings. The Sibling Violence sub-scale evaluates the individual’s experiences of physical and psychological violence indicted by their siblings. The Parental Violence sub-scale evaluates the individual’s experiences of physical and psychological violence from their parents or caregivers. The Interpersonal Violence sub-scale evaluates the witnessed behaviors of physical and psychological violence between parents or caregivers. Participants were asked to rate the frequency of experiencing the four types of maltreatment (variable reasoning, verbal aggression, and mild and severe physical tactics) between the ages of 6–12 and the ages of 13–18 on a 5-point scale (1 = never, 5 = every day). The internal reliability of each of the four types of relationships was excellent, with Cronbach’s alpha ranging from 0.93 to 0.97.

#### Community Violence Exposure (CVE).

A modified Richters and Martinez (Richters & Martinez, 1993) Things I have Seen and Heard sixteen-item scale was used to assess CVE. The items were on a Likert scale from never to many times (0–3) and were modified to add a single item querying exposure to seeing people having sex, based on previous work with this population (Burton et al., 2011). A total score and sub-scales for experiencing and witnessing were created.

#### Behavioral and Health.

##### Behavioral Health.

Participants completed four self-reported behavioral health functioning measures that included self-reported delinquency, self-esteem, and two measures of attachment style, and three validated mental health self-report measures that provide clinical diagnostic coding.

##### Self-Reported Delinquency.

Delinquency was measured with a 38-item version of the Self-Reported Delinquency Scales from the Denver Youth Study Crime and Delinquency protocol (SRD; Huizinga et al., 1991). Items assessed Delinquency based on the participants’ self-report descriptions of their delinquent activities, whether he/she ever committed an act, how many times in the past year, if others were involved, and if he/she was under the influence of alcohol or drugs while committing it, for each type of delinquent act. The 38 items were organized into sub-scales of public disorder, theft, violence, and financial crimes.

Self-esteem, attachment style and relationship were also assessed using the Coopersmith self-esteem inventory, relationship questionnaire, and relationship scales questionnaire respectively (see Supplemental Text 2)

#### Mental Health.

##### Youth Self Report.

The Youth Self Report is a self-report measure for youth ages 11 to 18. It is used to measure the emotional and behavioral issues of youth. The assessment itself consists of 112 items, with each item rated on a three-point scale. A 0 represents an absence of behavior, a 1 indicates it is somewhat true, and a 2 indicates that it is true very often. The reliability alpha in this study is between 0.7 and 0.93.

##### Beck Depression Inventory.

The Beck Depression Inventory is used to determine the severity of depression symptoms in youth. It is a self-report measure that has 21 items on a four-point Likert scale (Beck, 1967). By summing the scores from each component, a total score between 0 and 63 is calculated (Palmer & Binks, 2008). A score of 0–14 is termed minimal, 14–20 is mild, 20–29 is moderate, and 29–63 is severe. In this study, factors accounted for using this scale are performance impairment, somatic complaints, and general depression. Reliability in the current study yielded a Cronbach’s alpha of 0.4 to 0.85

##### Trauma Symptom Checklist.

The 42-item Trauma Symptom Checklist for Children (TSCC-A; Briere, 1996) was used to measure the prevalence of Post Traumatic Stress Disorder symptoms on a 4-point scale from 0 (never) to 3 (almost all the time). Two items assessing suicidality were removed for this study. Five sub-scales were created for post-traumatic stress symptoms (PTSS), anxiety, dissociation, anger, and depression. The internal reliability of each sub-scale ranged from 0.72 to 0.85. Clinical level of traumatic stress symptoms was set at 0–15 = Mild, 16–30 = Moderate, and 31 + = Severe.

### Executive Function and Cognitive Assessment

Participants completed a self-report of executive function, the Wisconsin Card Sort Task (WCST), four computerized cognitive assessments, and were evaluated individually on measures of current academic and intellectual functioning.

#### Executive Function.

The computerized battery included five executive functioning tasks: Go – No Go, Anti-Saccade, Task Switching, Flanker Shape, and Shape Matching. For each measure standard assessment of performance were calculated. The Go-No Go (GNG) task is a stop-signal response inhibition task with repeated button presses until participants receive a prompt to disinhibit that behavior (Nigg, 2000). The Anti-Saccade (AS; Friedman & Miyake, 2004) is a visual processing response inhibition task that requires oculomotor inhibition (Nigg, 2000). Task switching (TS) is a set switching task that requires the subject to switch mental sets between two different tasks (Friedman & Miyake, 2004; Rogers & Monsell, 1995). The Flanker Shape (FS) task requires interference processing, specifically resistance to distractor interference (Friedman & Miyake, 2004). The Shape Matching (SM) also requires distractor interference, but it required matching irregular shapes as well (Friedman & Miyake, 2004). Additional information about each of these tests can be found in the supplemental (see Supplemental Text 3)

Many studies remove poor performers from reporting as this can be a sign of failure to understand that task, unwillingness to follow instructions, or disruptions due to noise in the environment. For the purposes of the present study, a poor performance score was calculated and a count of poor performers generated. These poor performance indicators were then used to assess task performance in context and compare to disability diagnosis.

#### Cognitive Assessments.

##### Kaufman Brief Intelligence (KBIT-2).

The Kaufman Brief Intelligence Test Second Edition was used to evaluate verbal and non-verbal intelligence in participants (Carlozzi, 2011). The test consisted of questions involving verbal knowledge, matrices, and riddles.

##### Wide Range Achievement Test (WRAT-3).

The Wide Range Achievement Test 3 was used to evaluate participants’ academic skills in reading, mathematics, and spelling (Snelbaker et al., 2001).

##### Wisconsin Card Sort Task (WCST-CV).

Processing problems related to frontal lobe dysfunction, processing problems related to frontal lobe lesions, and learning disabilities were assessed using the Wisconsin Card Sort Computerized Version 4 (WCST-CV4; Heaton et al., 1993). The WCST-CV4 saved responses for each card trial and generated scores for how many trials it took for the subject to get a correct response, how often the subject persevered (or continued using an incorrect strategy), correct responses, errors, and a measure of how long it took the subject to learn a new pattern of responses.

##### Behavior Rating Scale of Executive Function (BRIEF-SR).

The 86-item Behavior Rating Inventory of Executive Function Self-Report Scale (Gioia et al., 2000) was used to evaluate self-reported executive functioning, including: Inhibit, Shift, Emotional Control, Monitor, Working Memory, Plan/Organize, Organization of Materials, and Task Completion. Sub-scales were combined into three indices according to the manual. Raw scores were converted to T scores and percentiles.

### Disability Diagnosis

Using eight academic and three mental health measures, where clinical diagnosis is possible, an overall number of super-clinical scores was calculated for each youth.

## Results

### Participants

The sample consisted of a convenience sample of 115 youth from a 200-bed facility where the daily census fluctuated but did not generally rise above 180. The 115 participants had a signed consent form returned, had signed an assent form, and were available for testing when researchers were there. There were 123 youth who both consented and assented but were not available for testing due to release from the facility, appointments, or behavioral restrictions.

### Record Review

From the medical records, the highest count of medical diagnoses was depression, asthma, antipsychotics, and ADHD. Around half of the incarcerated youth were on medications associated with depression. The criminal records indicated that the largest part of the sample was Class 2 and Class 3 sexual offenders (see Supplemental Text 4).

#### Educational Records.

Over 60% of youth had a prior Individualized Education Program (IEP), and there was evidence that over 62% had disability diagnoses (see Supplemental Table 2). The most prevalent special education disabilities in youth who have a history or currently have it are ADHD (41.5%), emotional disturbance (40.7%), and vision (47.2%). Other notable disabilities include cognitive disabilities (9.8%), speech (10.6%), communication (8.1%), and hearing (8.1%)

### Survey Methods

#### Demographics.

The sample consisted of 115 youth 13–20 years old and with between a 7th-grade and first year of college education (Author reference et al., 2011). Almost a third of the youth came from a two-parent family of origin, with close to 45% from single-parent families, the majority of those mother-headed households (see Supplemental Table 4). The sample was fairly evenly divided between races, with approximately one-third in white, black, and multiracial self-identification.

Nearly 90% self-reported previous locked detention, while over a third reported past placement in foster care, and nearly 20% previous residence in a group home. Previous psychological (85.3%) and substance abuse treatment (15.0%) were common (see Supplemental Table 5).

#### Violence Exposure.

Youth were asked whether they believed they had been sexually, physically, or emotionally abused with over 60% reporting abuse in each category (sexual abuse (SA) n = 69 (60.5%), physical abuse (PA) n = 72 (63.2%), emotional abuse (EA) n = 84 (73.7%). Just over 45% of youth reported that they had experienced SA, PA, and EA and 81.7% reported at least one type of abuse.

#### Adverse Childhood Experiences.

ACE total scores ranged from 0–12, out of 13 possible items, with a mean score of 4.65 (see Table 1). There were five youth (4.8%) who reported no ACEs, 30 (28.6%) who reported between one and three ACEs, and 70 youth who reported four or more ACEs (66.7%), which is considered a high exposure by most researchers.

#### Conflict

##### Tactics.

The CTS demonstrated a high frequency of overall IPV experienced and committed by the participants across childhood (M = 68.42, SD = 2.55). Each of the four subcategories of violence ranged from a score of 1 (no violence) to 5 (severe violence). The average violence score was approximately two in each category, corresponding to mild violence exposure.

##### Childhood Trauma Questionnaire.

Scores on the CTQ ranged from 1 to 5 in each subtype of childhood trauma (see Table 1). Due to the modification of the scale from the 28-item CTQ, mean scores, rather than sum scores, were compared to mean CTQ scores according to Bernstein and Fink (Bernstein et al., 1994), for Low, moderate, and Severe classification. The overall mean scores were at least low on emotional abuse (EA), physical neglect (PN), physical abuse (PA), and sexual abuse (SA), with a mean PA score of a moderate level. Three or more severe traumatic exposures were reported by 28 (22%) of the population and over 50% reported severe traumatic exposure on at least one subcategory of childhood trauma (n = 67). Between 25 and 41% of the population qualified for severe trauma on each of the five subtypes. At least 25% of the sample met criteria for Severe in all five subtypes of abuse. According to the cut-off scores, our data indicated that only 26% of the population reported no maltreatment in any of the five categories, while 12 (9.8% reported at least a low level of maltreatment on one scale, 21–22 reported low or greater maltreatment on two, three, or four scales, and 15 youth reported at least a low level of maltreatment on all five scales. The rate of PA, SA, rate of severe ACEs and rate of severe traumatic exposure were combined to examine the incidence of severe violence exposure in the population. Using these three measures 15%, 31% and 56% reported a severe level of violence exposure on one, two, or three of these measures.

##### Community Violence Exposure.

The average score on the Things I Have Heard and Seen Community Violence measure was 1.18, which is just over a mean of “once or twice” on a 0–3 scale with approximately the same mean for experienced and witnessed sub-scales (see Table 1). Both sub-scales and the total score produced high Cronbach’s alpha scores.

#### Behavioral Health.

##### Self-Reported Delinquency (SRD).

Chronbach’s alphas of the 37-item modified version of the Denver Youth Study SRD ranged from .64 - .92 (see Table 2). Mean endorsement of prior delinquency ranged from 31 times (violence) to 70 times (theft) in the last year.

The self-esteem, attachment style, and relationship scale questionnaire results can be found in the supplemental section (see Supplemental Table 6).

#### Mental Health.

##### Youth Self Report (YSR).

Means of all eight sub-scales of the Youth Self-Report were between 0 and 1, representing between “Not true” and “Somewhat or Sometimes True.” The presence of clinical levels of mental health problems is high for all eight sub-scales of the YSR. Between 12.2 and 45.2 percent of youth scored above the cut-off for borderline mental health on each of the eight sub-scales. Over forty percent of the population had a score above the clinical level on at least one sub-scale, with nearly 8% scoring at the super-clinical level on four or more sub-scales.

##### Beck Depression Inventory (BDI).

Means for the three sub-scales from the Beck Depression Inventory (BDI-II, General Depression (GD), Performance Impairment (PI), and Somatic Complaints (SC), were less than 1 on the 0–3 Likert scale. Over thirty percent of incarcerated youth reported at least mild depression. Notably, 17% of the youth had moderate depression symptoms. This means that the symptoms were noticeable and persistent.

##### Trauma Symptom Checklist for Children (TSCC).

Mean T-scores on the five sub-scales ranged from approximately 33 to over 80 when the clinical cut-off is a t-score of 65. Just over 20% of the sample was above clinical on at least one sub-scale of the TSCC.

### Executive Function and Cognitive Assessment

#### Executive Function.

The computerized battery contained five cognitive processing tasks, including two response inhibition tasks (Go - No Go (GNG) and anti-saccade task (AS)), Task Switching (TS), a cognitive flexibility test, and two interference processing tasks (Flanker Shape (FS) and Shape Matching (SM), see Supplemental Table 7).

#### Go – No Go (GNG).

Mean correct hits in the GNG trials was 55%. Correct rejections were higher, at 89%. However, correct hits and rejections were as low as 4% and 9% respectively. There were nine youth who were considered poor performers at under 30% correct hits or rejections.

#### Anti-Saccade (AS).

The percentage correct for the anti-saccade task was 54% and the RT was 771 ms. There were 21 youth whose percentage correct was below 25% (chance) or had an RT above 1000 ms.

#### Task Switching.

Mean TS scores were significantly higher during switching than the average reaction time (RT) of the non-switching tasks (*t*(98) = 18.220, *p* = .000). Mean Switch Cost (SC) was determined by subtracting the RT for switching from the average of RT for the non-switching conditions. Mean SC was 248 ms. Six participants were considered poor performers with a switch cost longer than 1000 ms.

#### Flanker Shape.

For the first interference processing task, the Flanker, RT were longer for interference and mixed trials than for control trials, and RT differed between control and interference trials (*t*(97) = 28.621, *p* = .000). There were nine participants who performed above the 750 ms cut-off point for the difference in RT between control and interference.

#### Shape Matching.

On the second interference processing task, the Shape Match, control RT were faster than interference and mixed RTs and the RT differed between control and interference (*t*(95) = 27.2348, *p* = .000). Seven participants had RTs greater than 500 ms, the poor performance cut-off. Thirty participants performed poorly on at least one indicator of executive performance.

#### Cognitive Assessments.

##### Kaufman Brief Intelligence (KBIT-2).

The KBIT-2 has standardized scores between 85 and 115 to be considered a nationally normed average. Most participants scored toward the lower end of the average range for all categories of verbal (*M* = 91.69, *SD* = 14.03), nonverbal (*M* = 96.77, *SD* = 14.43), and IQ (*M* = 93.95, *SD* = 14.54), with a grade equivalent range of 7th to 8th grade. The minimum scores for all three measures are in the lower extreme range, which is a nationally normed score of 69 or below. A total of 11.3% of participants meet verbal and/or non-verbal learning disability criteria (see Table 3).

##### Wide Range Achievement Test (WRAT-4).

Most participants scored slightly below average for all categories of the WRAT-3 (arithmetic (*M* = 84.53, *SD* = 15.27), reading (*M* = 89.24, *SD* = 15.78), and spelling (*M* = 90.23, *SD*= 15.99). Participants’ school grade equivalents for each skill are also slightly below average, with arithmetic being the lowest at about 7th grade (*M* = 6.85, *SD* = 3.28). Between 5.2% and 12.2% of youth score in the specific learning disability range.

##### Wisconsin Card Sort Task (WCST-CV).

Participant mean T-scores on the WCST-CV were around 45, which is the low end of the average (45–54, see Heaton 1991, see Table 3). T-scores of below 39 on this test are considered mildly impaired. Forty percent of youth had a T-score at the mildly impaired level or below in at least one of the five scores, with approximately 20% scoring at the mildly impaired level or below in all five main WCST indicators. Cronbach’s Alpha ranged from .75 to .94.

##### Behavior Rating Scale of Executive Function (BRIEF-SR).

The mean T-scores on the BRIEF-SR sub-scales were approximately 55, with a T-score above 65 considered clinically high. Approximately 25% of the population was in the clinical range for inhibiting and emotional control, with between five and twenty percent of the population above clinical on the other measures. Only 54% of the population were not above a T-score of 65 on any sub-scale, with 27% above clinical on two or more sub-scales. Inconsistency and negativity scores were low, indicating that most participant responses were consistent and responded to without an unusually negative manner (see Table 3). Cronbach’s Alpha ranged from .81 to .94.

### Disability Diagnosis

Using the three clinical measures (YSR, BDI, and TSCC), a total of 55.1% of the sample were above the clinical threshold for one mental health measure (see Table 2). Almost 20% of the participants were above clinical on one sub-scale of the YSR, BDI, or TSCC, and over 35% scored above clinical on at least two sub-scales.

Executive function (EF) tasks were included in the study to examine current cognitive functioning on tests of response inhibition, interference processing, and cognitive flexibility. Chi square tests were used to examine that poor performance on EF would be more common in participants diagnosed as having a disability using the limited measures used in the present study. Mental Health disability, as assessed by the YSR, BDI, and TSCC, was not associated with poor performance on EF (*χ*^2^(1) > = 0.137, p = 0.71).

Using only eight academic measures, a total of 77.6% of participants had at least 1 disability diagnosis (see Table 4). Between 1.2% and 10.6% of youth were found to have a super-clinical score within one domain of functioning. 22.4% had super-clinical scores on 2 scales and between 1.2 and 14.1% scored above the clinical level on measures within more than three domains. EF score and academic disability were not associated (*χ*^2^(1) > = 1.95, p = 0.16).

When the super-clinical counts were combined across mental health and academic functioning, nearly 85% of the participants were over clinical on at least one measure (see Table 5). Fourteen percent (14.2%) of the participants scored above the clinical cut-off on only one measure, while the other sixty percent scored above clinical by one, two, or more scales. There was no association between poor EF score and co-morbid disability status (*χ*^2^(1) > = 1.53, p = 0.2).

Finally, the overlap between disability and VE was examined using the *χ*^2^. Academic disabilities were more common in VE youth (*χ*^2^(1) > = 6.102, p = 0.014) but not mental health disabilities (*χ*^2^(1) > = 2.977, p = 0.084).

## Discussion

Multiple studies of adults who are incarcerated or former offenders have found that childhood violence exposure (VE;Ball, 2005; Jones et al., 2021) and both academic and mental health disabilities are common. However, there are fewer studies that examine either of these issues in incarcerated youth(Astridge et al., 2023) and even fewer that investigate VE and disabilities in the same youth sample, particularly with direct testing of current functioning. The current study was conducted to fill this gap and both test youth who are incarcerated for current cognitive, academic, and mental health diagnoses and survey the youth about VE. This represents an opportunity for understanding the challenges faced by this population, which is important for program development.

High levels of childhood violence exposure, childhood trauma, and disabilities were reported by both the youth self-report and through records search. It was found that youth with severe VE were more likely to have academic disabilities, but not mental health disabilities. Although it is not possible with the current study to understand direction of effect, prior work has shown that academic and cognitive disabilities are a risk factor for childhood abuse and neglect (Author reference, 2012)

Overall, nearly the whole population was diagnosed with an academic/cognitive or mental health disability (85%) using both individual testing and well-regarded clinical self-report measures. These high disability rates were primarily driven by cognitive and academic disabilities, which we found is approximately 75% of the youth.

The participants reported violence exposure at high rates, with about three-quarters reporting at least some trauma exposure, two-thirds qualifying for a high ACE score, and averaged experiencing family violence at least a couple of times a year. This combination of high exposure to prior violence and a vast majority with a diagnosable disability suggests that youth who are incarcerated come into the system with significant, but treatable, academic and mental health conditions. Knowledge of youth challenges upon entry into the juvenile justice system can help to develop targeted interventions for this population that can optimize the potential for reducing recidivism (Hall, 2015; Lockwood et al., 2012)

### Violence Exposure

Youth reported on average close to five previous experiences of ACE, where four or more is generally considered a high ACE score. Approximately two-thirds of these incarcerated youth reported at least four ACES, which is well above the average 18.5% of youth who experience four or more ACES (Swedo et al., 2024)A recent systematic analysis found that studies report between 11-72% of incarcerated youth report ACE and that the pooled estimate for this population is approximately 40%, similar to our findings (Astridge et al., 2023). Another systematic meta-analysis has reported that household dysfunction ranges from 10 to 36% of incarcerated male youth (Umpunjun et al., 2024) and between 12 and 38% on measures of child abuse and neglect.

Prior victimization was common according to other measures of violence exposure. The average youth reported on the CTS at least a couple of times a year of exposure to interpersonal, parental, and sibling violence, while reporting that they themselves were involved in family violence at least a couple of times a year. An early study of formerly incarcerated adults found that nearly 90% experienced at least mild physical abuse in childhood (Ball, 2005), which is similar to our findings in youth. In a study of incarcerated adult women, Jones and colleagues for a similar rate of experiencing IPV using the CTS; however since this was an adult population, this is partner victimization, not childhood exposure to IPV (Jones et al., 2021). Another Jones study of adult women found that perpetration of IPV was reported at a similar level (a few times a year) by both native and non-native women (Jones et al., 2021)

Reports of childhood trauma according to the CTQ were very high. Close to 75% of youth reported at least a low level of childhood trauma on one childhood maltreatment sub-scale of the CTQ. A minimum of 25% of youth reported a severe level of CM on each of the five measures, with over 40% reporting severe physical abuse and a third reporting severe sexual abuse. These rates a similar to the prevalence rates found by Umpunjun in their study of incarcerated males. A study from Australia found that nearly 40% of male youth met criteria for at least subthreshold PTSD using the CTQ; however, they reported much lower prevalence rates of severe scores in all five subscales (Moore et al., 2013).A more recent studies of youth in Switzerland (Heller et al., 2022) and China(Zhao, 2021)also reported lower rates of childhood trauma, however a Malaysian (Ahmad & Mazlan, 2014)and a different Australian study (Papalia et al., 2022) also found similar rates to ours with 52% of males reporting moderate to severe SA in the Malaysian study and 77% reporting at least one form of maltreatment, suggesting some variation cross-nationally and across study procedures.

Finally, exposure to community violence (CV) is known to be associated with violent offending (Hartinger-Saunders et al., 2011) and our population reported an average CV exposure of about once or twice a year according to the Richters and Martinez measure, which is higher than previous findings in at-risk youth (Thompson et al., 2007)

### Disabilities

Previous work has documented a link between disabilities and violent victimization in the general population (Author reference, 2012; Author reference, 2012)and a link in both incarcerated youth(Papalia et al., 2022)and adults (Jones et al., 2021).Although the current study cannot test causal direction, self-reported and record-reported disabilities prior to incarceration suggest that, regardless of causal direction, the combination of disabilities and childhood violence exposure is a major risk factor for youth offending behavior. In fact, given that over 60% of the population had a prior IEP or diagnosed disability via records search, this suggests that disability is a major risk factor for later youth offending behavior.

This study found high levels of mental health, cognitive, and physical disabilities of all kinds, both in the records review of pre-incarceration diagnosis and in the studied assessments. Similar to other research that found that disability doubles the risk of later incarceration (Mallet, the present study found that 60% of the youth had an IEP and/or diagnosed disability in their medical or educational records and found high levels of current cognitive deficits. During cognitive testing, we found rates of verbal, non-verbal, spelling, reading, and math deficits in between 3.5-30.6% of the population, much higher than expected in the general populations (Li et al., 2023) who found US rates of intellectual disabilities (ID) are around 1% and learning disabilities are 7%. This is also higher than rates found in a large study in Canada, where developmental disabilities were more than double in an adult prison population, but lower than the present study. (Whittingham et al., 2020).

Disabilities spanned cognitive and emotional. Learning and cognitive disabilities are well-documented in incarcerated populations and are considered a risk factor for offending. Bixby found that 60% of adults in federal prisons had non-psychiatric disabilities, similar to our pre-incarceration rate of IEPs and to the rate of concurrent non-psychiatric clinical level scores we found in our population. This study is fairly unique in reporting individual cognitive and academic testing in an incarcerated youth population, and we were able to confirm through our own testing that the youth had high levels of cognitive disabilities. Just over 10% of the population scored at below the cognitive delay cut-off on the KBIT-2, which is far higher than the national average (Li et al., 2023).Similarly, the population exhibited prevalence rates of learning disabilities that are far higher than national averages at 17-30% of the population. at 17-30% of the population. Nearly 45% of the population tested above the clinical threshold for executive functioning, as measured by the BRIEF-SR, and just over 40% tested at a level considered at least minimally impaired on the WCST, an indication of potential cognitive injury (Heaton et al., 1993).This rate far exceeds the percentage of adults with impairment, or 14.6% in the original normal study.

Impaired scores on the WCST are known to be common in brain-injured populations, suggesting that undiagnosed brain injury may be common in this population. Our educational records search found that approximately four percent of our population had an IEP based on neurological disability, which suggests that at least some had a history of traumatic brain injuries (TBI). This rate is higher for neurological problems in youth (Banerjee et al., 2009).Although the present study did not have a direct measure of brain injury, a study of Australian detainees found that 33% reported head injury. While Davies and colleagues (Davies et al., 2012) found that more than 70% of their participant pool of incarcerated youth had experienced at least one head injury, and 41% experienced some loss of consciousness, indicating that TBI may be a common correlate with violent offending, either cause or effect. Our findings from the WCST are consistent with this range of one to two-thirds of incarcerated populations showing signs of TBI.

Another study reported that youth with high rates of epilepsy symptoms, head trauma, or loss of consciousness were much more likely to be sent to juvenile training school than comparison youth (Miura et al., 2005) .In the present study we find support for high rates of seizure activity, another potential indicator of previous head trauma. Few studies have examined neuro-psychological function in incarcerated youth or adults, but some have found that impaired scores on the WCST are associated with anti-social and aggressive Behaviors (Delfin et al., 2018). Taken together, our study indicates a pervasive problem with cognitive disabilities in youth who are incarcerated.

Our BRIEF-SR findings indicate that executive function was common, a finding that was supported by our cognitive functioning battery. With a 40% rate of clinical executive functioning deficit, this work is consistent with prior work that shows that juvenile offenders had high impulsivity, more attentional bias, and lower executive function in various cognitive tests (Patiz & Bayraktar, 2023).Furthermore, in another study, youths with shorter periods of detention had higher cognitive performance, while longer captivity correlated with lower executive functioning and high impulsivity (Bauman et al., 2023)suggesting that lower EF is a risk factor for more serious offending. Our medical record search and mental health testing also support the findings that impulsivity are a problem for this population, with 28% having a previous diagnosis of ADHD and approximately 20% showing at least borderline clinical levels of attention problems with the YSR.

There was a very high rate of mental health diagnoses in the population as well. Approximately 20% of the population had moderate or severe depression according to the BDI-II. This is lower than a recent study which showed 25% had moderate depression, and 22% had severe depression (Domalanta et al., 2003).However, our study found that close to 45% of the population had at least one category where they scored above clinical on the YSR. Our sub-scale rates are compared to a recent study from Switzerland that found that incarcerated youth have a prevalence rate of 26.1% for externalizing disorders (ADHD, conduct disorders, and opposition disorders), and a prevalence rate of 18.8% for internalizing disorders (depression and anxiety; Heller et al., 2022). Our results are slightly higher than a recent Australian study that used the YSR and found rates of mean incidence rates on the YSR between 4 and 16%, whereas out sub-scale clinical incidence rates are between 10 and 27%.

Comorbidity of diagnosis was common with around 80% of the youth having at least one clinical level score on a diagnostic instrument. This is consistent with prior work from the last century documenting at least one diagnosis in 72% of youth in a small study of incarcerated youth (Atkins et al., 1999)and two more recent studies, one of youth in temporary detention where the rate for males was 66% (Teplin et al., 2002), and one of incarcerated youth with an 83% rate at least one psychiatric diagnosis(Heller et al., 2022). A more recent study from Canada found over a 90% rate of at least one diagnosis in incarcerated youth (Gretton & Clift, 2011), and one from the US found a rate of 70% in incarcerated adults in federal prison (Bixby et al., 2022),similar to our findings.

Our work suggests that comorbid diagnosis is predominately driven by high rates of cognitive disabilities. Only 22% of the population did not fit criteria for diagnosis with an academic disability using the KBIT-2, WRAT-4, BRIEF-SR, and WSCT-CV. The largest group of youth who reported only one diagnosis exhibited either executive function or frontal lobe deficits (according the BRIEF-SR and WCST-CV, respectively).

Approximately 35% of the youth exhibiting a diagnosable level of academic functioning on two or three measures. This is higher than the Heller study (Heller et al., 2022)that found over 20% of youth with 2 or 3 comorbid diagnoses. More than 40% of the whole population showed evidence that they could be diagnosed with a minimum of two comorbid academic conditions, which is over 50% of the youth with a diagnosable academic condition. When mental health comorbidity is added, over 60% of the population was found to have more than one clinical level score. Few other studies have examined comorbidity in incarcerated youth, particularly when individual testing is conducted.

Incarcerated youth have significantly different health profiles than those of the same age. According to Forrest et al (Forrest et al., 2000),incarcerated youth had significantly worse health compared to their counterparts in the domains of self-esteem, physical discomfort, acute disorders, chronic disorders, and psychosocial disorders. The worst health profile described 69.8% of the sample of incarcerated youth compared to the 30% in an age-matched school sample (Forrest et al., 2000). Only 6.4% of the incarcerated youth were in the good/excellent health profile, which was 34.2% of the school sample. Our own study showed particularly high rates of seizures, asthma, and bed wetting. One bright spot in the study is relatively high self-esteem and strong attachment. This suggests that incarcerated youth have the basic skills necessary to harness the neural plasticity of youth.

Prior theoretical work on the social causes of juvenile delinquency suggests that violent victimization, including VE, is a major factor is creating general strain (Broidy, 2001)as a cause of violent perpetration. Although our own work does not test the direction of effect, future research should focus on the role that comorbid disability and VE in predicting juvenile delinquency. Recent General Strain theory work highlights the role of teacher punishment (Moon et al., 2008) and bullying (Cullen et al., 2008)as particular social strains that predict perpetration. Both teacher punishment (MacSuga-Gage et al., 2021)and bullying (Bear et al., 2015) are more common in youth with disabilities, suggesting that juvenile justice theories may be missing the role that prior cognitive and academic challenges play in juvenile perpetration (Mueller et al., 2019).

### Policy and Clinical Implications

One important finding from the current study is that youth who are incarcerated have high rates of both academic and psychiatric diagnoses and VE; however, the direction of effect is unclear. One implication of the research is that youth entering detention should be tested for cognitive impairments of various kinds. Prevention of violent offending cannot always be attained; however, when youth are convicted, this is a potential inflection point for intervention. Strong academic, cognitive, and trauma-informed clinical interventions are necessary for the growth and development of this population while in the care of the state. As a policy, youth academic growth in youth incarceration facilities should be closely tracked. Large gains in academic functioning in this population should be observed with strong academic intervention. There is work that shows that educational improvements decrease offending behavior, and this is an excellent time for targeted educational interventions.

### Social Justice Implications

Incarcerated youth are a special population that is understudied, and, often due to constraints on working in youth incarceration facilities, there are even fewer studies that obtain data from multiple sources. Inclusion of youth from facilities of all times is important for broader inclusivity in research. Through considering the participants’ experiences with adversity and exposure to violence, the study was able to identify cognitive disparities through the lens of social justice and inclusivity. Using medical records, surveys, and cognitive testing, we were able to identify the health disparities in this population and the need for continued inclusive research within this population. The results of this study inform solutions to health equity in the future.

### Future Directions

Future research should examine subgroups of high violence and disabilities and their aggression and offending behavior. Of particular interest is head injury in this population. It would be interesting to examine the relation that WCST clinical level of functioning has with violence exposure in this population. That may give an indication of whether prior violence exposure could be considered causal, or at least connected, to evidence of TBI.

Future research should examine the relation between performance on test of cognitive function (such as the WCST), violence history, and mental health outcomes, such as aggression. Previous work has shown that prior VE is associated with offending behavior; however, it is unclear if disability is a mitigating factor.

## Supplementary Material

Tables 1 to 5 are available in the supplementary files section

This is a list of supplementary files associated with this preprint. Click to download.

CDDescriptivesSupplementalTablesMethods.docx

Tables.docx

## Data Availability

Data is available through Perkins, S. C., Smith-Darden, J., & Graham-Bermann, S. Cognition and Disabilities in Incarcerated Male Youth. Ann Arbor, MI: Inter-university Consortium for Political and Social Research [distributor], 2023-07-12. https://doi.org/10.3886/E147341V1
